# Clinical and Mechanistic Review of Amiodarone-Associated Optic Neuropathy

**DOI:** 10.3390/biom12091298

**Published:** 2022-09-14

**Authors:** Reece Mitchell, Joseph Chacko

**Affiliations:** 1 College of Medicine, University of Arkansas for Medical Sciences, Little Rock, AR 72205, USA; 2 Department of Ophthalmology, University of Arkansas for Medical Sciences, Little Rock, AR 72205, USA

**Keywords:** amiodarone, optic neuropathy, non-arteritic ischemic optic neuropathy

## Abstract

Amiodarone-associated optic neuropathy (AAON) is a complex clinical diagnosis, requiring distinction from non-arteritic ischemic optic neuropathy (NAION) due to a shared at-risk patient population. Diagnosis of AAON is complicated by a varied clinical presentation and incomplete pathophysiologic mechanisms. This article reviews pertinent literature for describing and clinically delineating AAON from NAION, as well as newly reported protective mechanisms of insulin-like growth factor 1 (IGF-1) and PI3K/Akt against amiodarone-induced oxidative and apoptotic injury in retinal ganglion and pigment epithelial cells. These studies offer a basis for exploring mechanisms of amiodarone toxicity in the optic nerve.

## 1. Amiodarone Pharmacology and Toxicity

Amiodarone is a class III antiarrhythmic agent with characteristics of class I, II and IV antiarrhythmics, and it is widely prescribed for the management of atrial fibrillation and ventricular fibrillation/tachycardia [1]. Amiodarone has been increasingly utilized over strict class I antiarrhythmics due to decreased proarrhythmic toxicity [1]. It has also been shown to significantly reduce mortality of sudden cardiac death [2]. Amiodarone is one of the most effective antiarrhythmic drugs for treating supraventricular and ventricular arrhythmia; however, clinical usage varies widely due to the need for consistent patient monitoring.

Pharmacologically, amiodarone distributes to all major body compartments, with adipose tissue acting as a reservoir due to high lipid solubility [3]. Its oral bioavailability ranges from 30% to 80%, with half-life ranging from 20–100 days [4]. Due to variations in half-life and adipose reservoir between patients, cumulative daily dosing and treatment duration are most effective for clinical monitoring compared to plasma drug levels. Amiodarone is metabolized through hepatic cytochromes CYP3A4 and CYP2C8 into an active metabolite, desethylamiodarone (DEA). Both drugs are predominately eliminated by biliary excretion, and dosing changes are unnecessary in patients with renal insufficiency [5]. Amiodarone and DEA inhibit several cytochrome enzymes and can cause significant drug–drug interactions [6].

The clinical utility of amiodarone is further hindered by significant side effects, including hypersensitivity pneumonitis, thyroid dysfunction, photosensitivity, neuropathy, and ocular changes [7,8,9,10,11]. Numerous case reports and series have linked amiodarone usage to optic neuropathy [12,13,14]. Approximately 50% of patients on long-term use eventually discontinue amiodarone due to notable side effects and risk of serious complications. Verticillate keratopathy is the most frequently observed ocular change, occurring in 70% to 100% of patients on amiodarone therapy [15]. Amiodarone-associated hypersensitivity pneumonitis is a particularly fatal complication of long-term amiodarone usage that requires close monitoring.

## 2. Clinically Defining Amiodarone-Associated Optic Neuropathy

Amiodarone-associated optic neuropathy (AAON) is a clinical diagnosis with significant variation in presentation, which makes individual diagnoses and establishing objective diagnostic criteria for AAON particularly challenging. Additionally, AAON requires distinction from non-arteritic ischemic optic neuropathy (NAION), the most common cause of optic nerve-derived vision loss in individuals above age 50 [16]. The exact mechanisms of NAION are unproven, but the presumed pathogenesis relates to transient disruptions to optic nerve circulation from hypoperfusion, thrombosis, venous occlusion, or vasospasm [17]. Individuals with a small cup-to-disc ratio may be predisposed to NAION, because optic disc edema may lead to easier vascular compression and secondary ischemia at the rigid lamina cribrosa [18]. The resultant ischemic process leads to visual field defects, with arcuate and inferior altitudinal defects being most common [18]. Importantly, persons with high-risk cardiovascular disease on amiodarone therapy are at increased risk for both AAON and NAION, further necessitating reliable clinical criteria for AAON. Macaluso and colleagues provide a framework for distinguishing AAON from NAION, including insidious onset, slow progression, bilateral vision loss, and protracted disc edema over multiple months [19]. In contrast, NAION develops rapidly within days to weeks with unilateral involvement, and disc edema resolves within weeks. The reported decrease in visual acuity with AAON tends to progress from 20/20 to 20/200, whereas NAION often results in decreases in acuity from 20/20 to no light perception [19].

Using this diagnostic basis, Purvin et al. classified 7 of 22 patients who developed optic neuropathy while taking amiodarone with either AAON (4) or NAION (3), excluding other possible systemic causes [20]. Because 15 undifferentiated patients exhibited characteristics of AAON and NAION, additional recommendations were proposed: patients with unilateral disc edema were differentiated based on typical or atypical features of NAION, while patients with bilateral disc edema were evaluated to exclude other systemic causes of optic neuropathy, including pseudotumor cerebri and giant cell arteritis. Atypical features of NAION were defined as having insidious onset, mild optic nerve dysfunction (qualitative visual field assessment, a visual acuity ≥20/40, and a relative afferent pupillary defect [RAPD] of ≤0.9 log units), a generous cup-disc ratio in the opposite eye, and a prolonged duration of disc edema [20]. Typical features of NAION were defined by the same features reported by Macaluso et al. [19]. NAION is characterized by a crowded fellow disc, so its absence should create doubt in diagnosis. Additionally, the presence of systemic symptoms of amiodarone toxicity increased suspicion for AAON. All patients with simultaneous bilateral disc edema (14) were classified with AAON, despite variations in duration of disc edema and onset of visual symptoms. Patients with unilateral disc edema and typical features of NAION (3) were all classified as having coincidental NAION unrelated to amiodarone therapy. Five patients with unilateral disc edema and one or more atypical features of NAION were classified as having an indeterminant mechanism of optic neuropathy.

## 3. Refining Diagnostic Criteria for AAON

In 2017, Fasler et al. applied the diagnostic framework reported by Purvin et al. in a retrospective chart review of 13 patients with suspected optic neuropathy related to amiodarone therapy between 1995 and 2015 [21]. Exclusion criteria included other plausible causes of optic neuropathy and incomplete information related to amiodarone therapy and dosing. Interestingly, 3 patients were identified as belonging to a newly defined subclassification for differentiating AAON versus NAION, in which sequential optic disc swelling occurred as opposed to a strict presentation of either bilateral or unilateral disc edema. The interval to involvement of the second eye ranged from 3 to 48 weeks. Each patient with sequential disc edema (3) exhibited at least one atypical feature of NAION, thereby lending support to the existence of sequential eye involvement in AAON progression. All other patients were classified with bilateral edema (6) or strict unilateral edema with either typical (1) or atypical (3) features of NAION. In each case of bilateral edema, at least one additional atypical feature of NAION was present, including insidious onset and visual acuity ≥ 20/40. Diagnosis of AAON or NAION for patients with strict bilateral or unilateral disc edema was made similarly to Purvin and colleagues.

## 4. Persistent Diagnostic Challenges for AAON

Despite the development, refinement, and application of diagnostic criteria for AAON through the aforementioned studies, AAON remains a controversial diagnosis with unclear pathophysiologic mechanisms and challenging clinical distinction from NAION. To date, Mindel et al. are the only researchers who have performed a prospective, controlled, double-blinded approach to identify incidence, dosing, and time until onset of bilateral vision loss from amiodarone [22]. Using over 1600 randomized subjects receiving either body-weight dependent amiodarone dosing or placebo over a median administration period of 45.5 months, they failed to identify and remove a single subject from the study due to bilateral vision loss. Although ophthalmologic examinations were not performed during the study due to concern for unmasking pathognomonic signs of AAON like verticillate keratopathy, no reports of bilateral vision loss occurred during the minimum observation period of 27 months. In the absence of reported bilateral vision loss, binomial 95% CI calculations determined maximum possible annual incidence rates of AAON for each category of weight-adjusted dosing groups: > 2.0 mg/kg (n = 696), > 3.0 mg/kg (n = 559), or > 4.0 mg/kg (n = 219) had maximum possible (95% confidence) annual incidences of bilateral toxic vision loss of 0.23%, 0.29%, or 0.74%, respectively [22]. These calculated incidences conflict with many previously reported retrospective incidences ranging from 0.36% to 2% [8,23,24]. The prospective findings from Mindel et al. cast debate over whether AAON exists as a clinically distinct etiology of optic neuropathy or merely a variation in presentation of NAION.

## 5. Investigating AAON Incidence

A more recent retrospective report by Cheng et al. sought to clarify amiodarone usage and increased risk of optic neuropathy [25]. Using Taiwan’s National Health Insurance Research Database (NHIRD), 6175 individuals were identified as being newly treated with amiodarone between 2005 and 2009. For each amiodarone-treated patient, four age and gender matched control patients (24,700) were included. Pertinent data regarding medical comorbidities including hypertension, diabetes mellitus, coronary artery disease, cerebrovascular disease, obstructive sleep apnea, cancer, and chronic kidney disease were recorded for both groups. Optic neuropathy was successfully identified in 17 amiodarone-treated patients (0.3%) and 30 control patients (0.1%) using the following criteria: a documented optic neuropathy diagnostic code, visual field exam on the day of diagnosis, and a fundus examination of any type. Data related to amiodarone daily dosing and treatment duration was also collected to explore the effect of cumulative amiodarone dosing and developing optic neuropathy.

Using univariate and multivariate Cox analysis, Cheng et al. demonstrated that amiodarone patients had a 2-fold increased risk of optic neuropathy with a hazard ratio of 2.09 (95% confidence of 1.13 to 3.85, *p* = 0.02) after adjusting for age, gender, and medical comorbidities. While amiodarone treated patients had a significantly higher proportion of medical comorbidities, only amiodarone usage and hypertension were found to be significant risk factors for optic neuropathy in males but not among females. For females, only cancer had a significant risk association with optic neuropathy. Amiodarone usage and other medical comorbidities had no observed significant risk association for optic neuropathy in women. Among amiodarone-treated patients, male gender was associated with a 3-fold increased risk of developing AAON, and this conclusion agrees with prior research findings [26]. Multiple factors may contribute to this observed discrepancy in AAON incidence between males and females, including gender-related differences in body mass and fat distribution, CYP enzymatic activity, and hormonal stimuli [27]. When examining the effect of treatment duration on developing optic neuropathy, the average duration of treatment for patients who developed optic neuropathy was significantly longer (111.7 days) than patients without optic neuropathy (71.6 days; *p* = 0.03). This significance remained consistent when comparing optic neuropathy incidence in patients with treatment durations above and below the median of 41 days, and this treatment duration has been shown in a prior meta-analysis to increase risk for optic neuropathy by 3.5-fold [28]. Interestingly, the average daily amiodarone dose was not significantly different between the two groups (177.1 mg/day with optic neuropathy vs. 245.4 mg/day without optic neuropathy; *p* = 0.15). This lack of significance in amiodarone dosing remained consistent when comparing above and below the median dose of 200 mg/day, as well as across tertiles (≤190, >190–39, >239 mg/day). Lafuente-Lafuente et al. showed that amiodarone concentrations in adipose tissue were not correlated to daily amiodarone dose [29], thereby reinforcing cumulative dosing and treatment duration rather than daily dosing as the clinical standard for evaluating risk of AAON and other systemic toxicities in patients receiving amiodarone.

## 6. Early Mechanistic Investigations of AAON

Much of the uncertainty and debate surrounding the incidence and degree of distinction of AAON from NAION is due to a lack of understood pathophysiologic mechanisms of amiodarone toxicity on the optic nerve. Early ultrastructural investigations revealed lysosomal penetration by amiodarone, leading to interference with cellular metabolism [30]. Subsequent histologic analysis of ocular tissue samples revealed intracellular membranous lamellar inclusion bodies in asymptomatic patients treated with amiodarone, and these inclusions are hypothesized to disrupt axoplasmic flow and contribute to drug-induced lipidosis within large axons of optic nerve cells [31]. This impairment of axoplasmic flow may contribute to optic disc swelling observed in AAON. Additional studies have demonstrated toxic effects of amiodarone, including increased release of inflammatory mediators, mitochondrial dysfunction, and free-radical formation [32]. Amiodarone has also been shown to induce mitochondrial H_2_O_2_ synthesis and the formation of reactive oxygen species (ROS), which cause oxidative cellular damage [33,34].

## 7. Retinal Pigment Epithelial Cell Models of Amiodarone Toxicity

**Figure 1:**
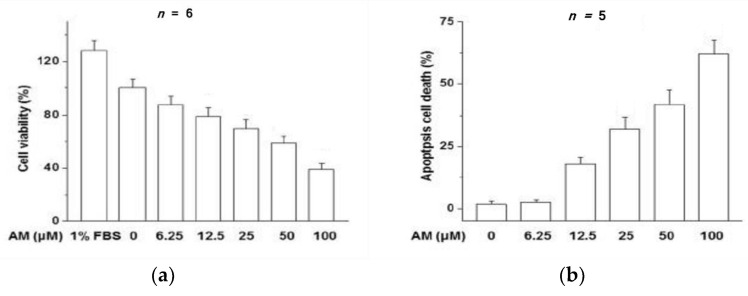
(**a**) The percentage of viable RPE cells decreases from 100% to approximately 40% as amiodarone (AM) concentration increases from 0 μM to 100 μM. (**b**) The percentage of RPE cells undergoing apoptosis increases from 0% to approximately 63% as AM concentration increases from 0 μM to 100 μM [37].

**Figure 2:**
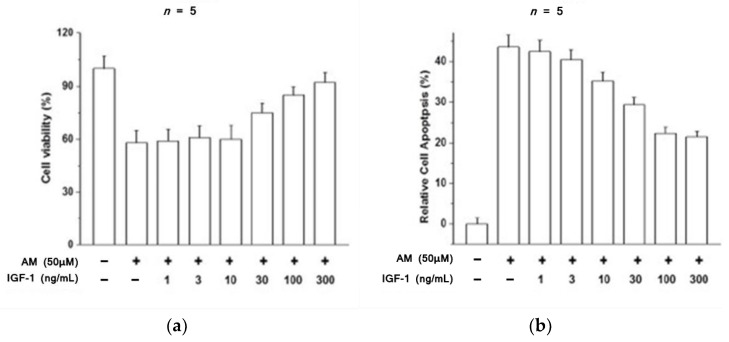
(**a**) The percentage of viable RPE cells decreases dramatically with the introduction of 50 μM AM, but this decline is attenuated by increasing the concentration of administered IGF-1 from 1 ng/mL to 300 ng/mL. (**b**) The percentage of apoptotic RPE cells significantly increases with the addition of 50 μM AM. The effect of AM on apoptosis is attenuated by addition of IGF-1 over a concentration range of 1 ng/mL to 300 ng/mL [37].

While direct investigations into optic nerve toxicity through amiodarone-induced oxidative injury have yet to be performed, closely related studies of the retinal pigment epithelium (RPE) have successfully described protective cellular mechanisms against amiodarone toxicity. The RPE is formed from a single layer of polygonal cells at the outer edge of the retina. The RPE utilizes microvillous structures to phagocytose exfoliated photoreceptor cell outer segments, which help maintain the renewal of visual cells [35]. Additionally, the RPE absorbs light via melanin and lipofuscin pigment, thereby protecting neural components of the retina from oxidative damage through antioxidants like glutathione and superoxide dismutase. Insulin-like growth factor 1 (IGF-1) has been previously identified as a promoter of cell survival against oxidative stress [36]. Liao and colleagues showed that IGF-1 and the ubiquitous PI3K/Akt pathway are key mediators in protecting RPE cells from amiodarone associated oxidative stress [37]. Liao et al. demonstrated a concentration-dependent effect of amiodarone on decreased RPE viability and increased apoptosis, with an LC_50_ = 50 μM (Figure 1). From this basis, they showed that IGF-1 promoted RPE cell survival from amiodarone-induced oxidative toxicity through a MTT assay and Hoechst DNA staining technique (Figure 2). To distinguish which downstream IGF-1 signaling pathway provided cellular protection from amiodarone toxicity, a series of MAPK and PI3K/Akt pathway inhibitors were added to RPE cells treated with amiodarone.

RPE cells were exposed to amiodarone (50 μM) in the presence of the PI3K inhibitor LY294002 (30 μM), the Erk MAPK inhibitor PD98059 (25 μM), or the p38 MAPK inhibitor PD160316 (10 μM), and then stimulated with IGF-1 (100 ng/mL). Inhibition of the MAPK pathway showed no effect on the ability of IGF-1 to protect against amiodarone toxicity, and PI3K inhibition causes a decrease in the protective effect of IGF-1 against apoptosis.

**Figure 3:**
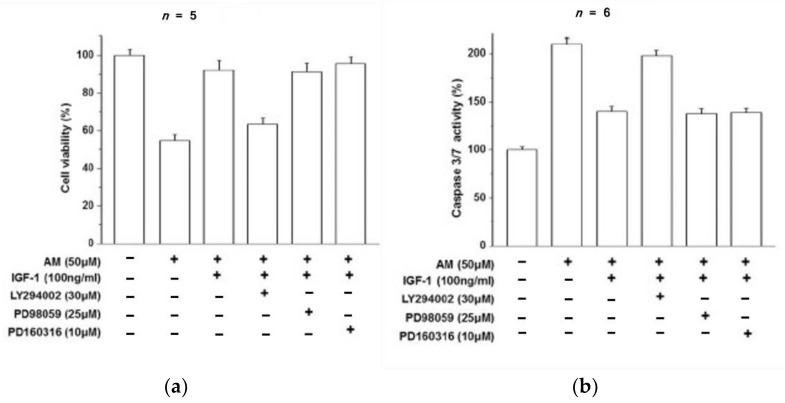
(**a**) The percentage of viable RPE cells decreases in response to AM exposure and is protected by IGF-1. Inhibiting the PI3K/Akt pathway by LY294002 causes a reduction in IGF-1 efficacy, while inhibiting MAPK by PD98059 or PD160316 has no effect on maintaining RPE cell viability by IGF-1 in the presence of AM. PI3K/Akt is the pathway by which IGF-1 protects RPE cells from AM toxicity. (**b**) Caspase 3/7 activity in RPE cells approximately doubles in the presence of AM compared to control, and IGF-1 attenuates this increase. Inhibiting the PI3K/Akt pathway by LY294002 diminishes apoptotic protection from IGF-1, while inhibiting MAPK by PD98059 or PD160316 has no effect on the ability of IGF-1 to reduce caspase 3/7 activity in the presence of AM [37].

**Figure 4:**
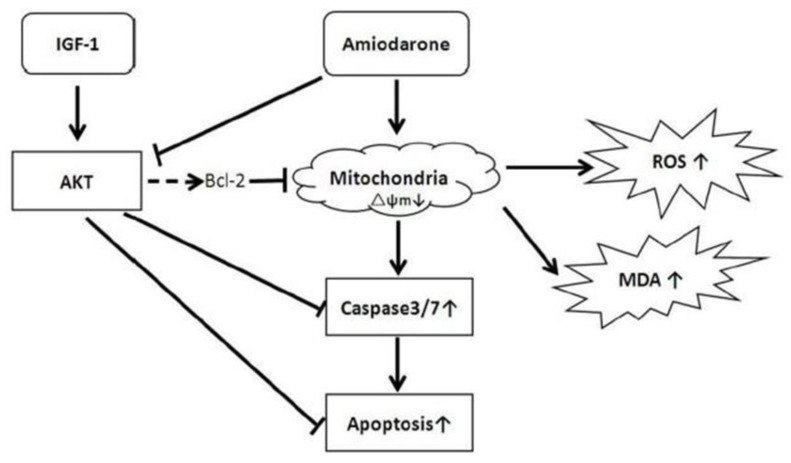
A proposed mechanism of IGF-1 mediated attenuation of amiodarone toxicity in the RPE. Amiodarone inhibits the activity of Akt and decreases mitochondrial membrane potential stability (∆ψm). This membrane instability in turn results in increased ROS production, MDA lipid peroxidation, and caspase 3/7 driven apoptosis. IGF-1 stimulates the activity of Akt, thereby reducing oxidative and proapoptotic mechanisms of cell death from amiodarone [37].

Thus, activation of the PI3K/Akt pathway was necessary for inducing the protective effects of IGF-1 against amiodarone toxicity (Figure 3). Mechanistically, amiodarone blocked phosphorylation of Akt while IGF-1 stimulated Akt phosphorylation, thereby enabling the protective PI3K/Akt pathway to mitigate oxidative injury from amiodarone. Subsequent experiments showed that IGF-1 had additional protective effects on amiodarone-treated RPE cells. IGF-1 reduced ROS and lipid peroxidation of malondialdehyde (MDA), helped maintain the mitochondrial membrane potential (∆ψm), and inhibited apoptotic caspase 3/7 activation (Figure 4).

## 8. Retinal Ganglion Cell Models of Amiodarone Toxicity

**Figure 5:**
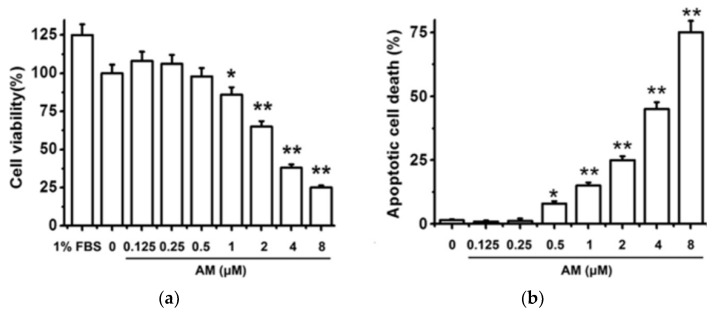
Amiodarone (AM) decreases cell viability and increases apoptosis in a concentration-dependent manner. (**a**) RGCs were treated with amiodarone and measured for cell viability using an MTT assay at 24 h. (**b**) Cellular apoptosis was determined using Hoechst 33342 staining at 24 h. The data are expressed as the mean ± SEM of results obtained from five independent experiments. * p < 0.05, ** p < 0.01 [40].

In addition to RPE cell models, IGF-1 has also been shown to protect retinal ganglion cells (RGCs) from death and encourage axonal regeneration [38]. RGCs function to transmit visual information derived from photoreceptors and interneurons through the optic nerve and onto the brain for higher order visual processing [39]. Additional investigation by Liao et al. demonstrated the ability of IGF-1 to specifically protect against amiodarone-induced apoptosis in RGCs [38]. Amiodarone had a concentration-dependent effect on decreased RGC viability and increased apoptosis at submicromolar concentrations with a LC_50_ = 2 m (Figure 5).

**Figure 6:**
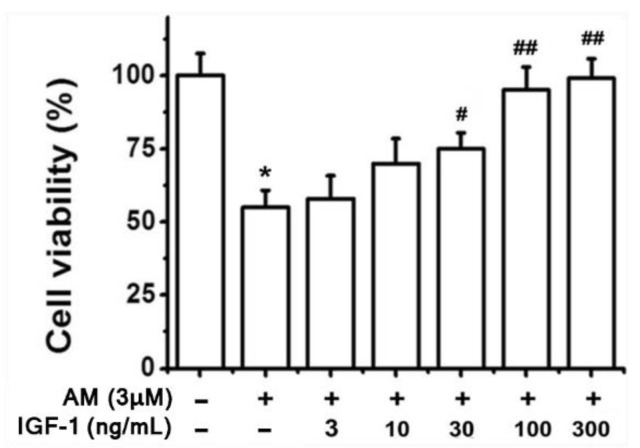
IGF-1 pretreatment preserves RGC viability in the presence of AM. RGC cells received varying concentrations of IGF-1 and then exposed to 3 μM AM for 24 h. Cell viability was determined via the MTT assay. Results show the mean ± SEM of five independent experiments. * p < 0.05 compared with control, *# p* < 0.05 compared with amiodarone, *## p* < 0.01 compared with amiodarone [40].

**Figure 7:**
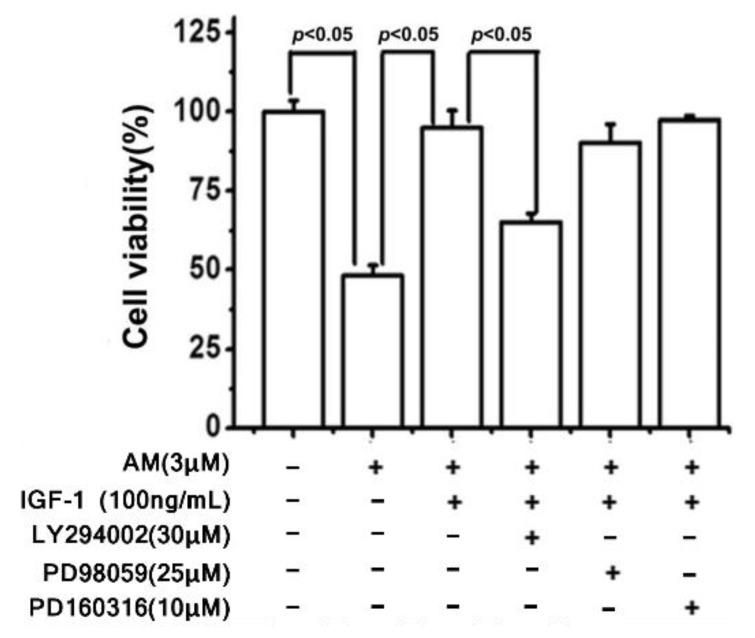
LY294002, a PI3K inhibitor, inhibited the protective effect of IGF-1 against amiodarone-induced apoptosis of RGC cells. The ERK pathway inhibitor PD98059 and p38 MAPK inhibitor PD160316 had no effect. RGC cells were pretreated with either LY294002, PD98059, or PD160316 and then treated with IGF-1 and amiodarone for 24 h. Cell viability was determined by the MTT assay. Data are expressed as a percentage of the corresponding control value which was normalized as 100%. Results show the mean ± SEM and represent assays from five independent experiments [40].

Given the smaller LC_50_ of RGCs compared to RPE cells, RPE cells are significantly less sensitive to amiodarone toxicity when comparing proportions of the cell culture undergoing apoptosis [37]. The observed discrepancy between RGC and RPE amiodarone sensitivity is likely related to the antioxidative function of PRE cells, thereby conferring greater resistance to amiodarone-induced toxicity. IGF-1 pretreatment was then shown to preserve RGC viability in the presence of 3 μM amiodarone (Figure 6). The preservation of RGC viability from amiodarone toxicity through IGF-1 agrees with similar findings for RPE experiments (Figure 2a). However, the concentration of amiodarone needed to observe the protective effect of IGF-1 varies considerably due to cellular antioxidative characteristics. The same series of PI3K/Akt and MAPK inhibitors described in Figure 3 were applied to RGC cells pretreated with IGF-1 and exposed to amiodarone. Again, PI3K/Akt was the downstream signaling pathway of IGF-1 that provided RGC protection from amiodarone (Figure 7).

**Figure 8:**
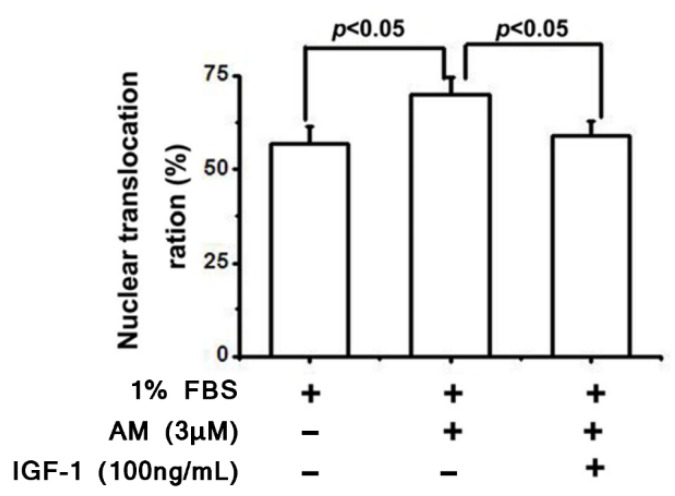
Amiodarone increases nuclear translocation of FoxO3a in RGCs, leading to increased apoptosis. IGF-1 significantly attenuates this process in amiodarone-treated RGCs. Fluorescence microscopy was utilized to identify cytosolic or nuclear localization of FoxO3a, and the nuclear translocation ration was calculated as the percentage of RGCs with nuclear predominance of FoxO3a compared to all cells that could be classified as either nuclear or cytosol predominant [40].

**Figure 9:**
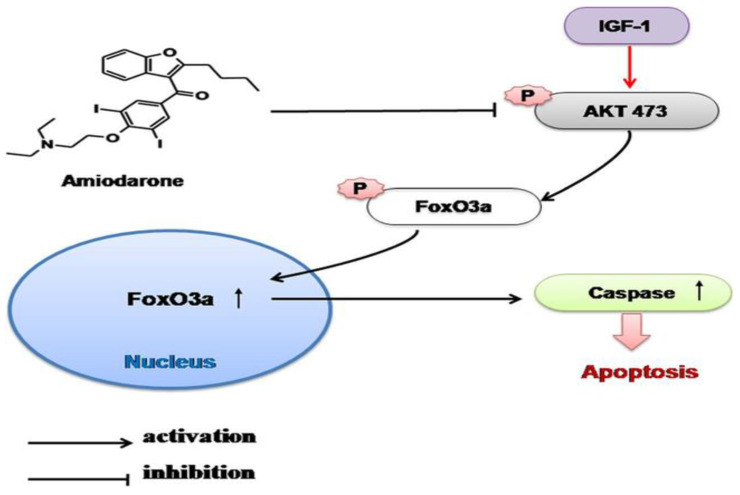
A proposed mechanism of IGF-1 and amiodarone in the RGC. Amiodarone decreases Akt phosphorylation of FoxO3a, leading to nuclear translocation of FoxO3a and increased caspase mediated apoptosis. IGF-1 antagonizes amiodarone by stimulating Akt phosphorylation of FoxO3a, leading to increased cytosolic localization and improved RGC survival [40].

As a final mechanistic investigation, Liao et al. explored the cellular distribution of FoxO3a, a downstream transcription factor of PI3K/Akt. FoxO3a is phosphorylated by Akt, thereby preventing nuclear translocation and activation of apoptotic pathways. By introducing amiodarone to RGCs, the proportion of FoxO3a translocated to the nucleus increased, and this effect was successfully attenuated by pretreatment with IGF-1 (Figure 8 and Figure 9).

## 9. Conclusions

While RGCs and RPE cells are not entirely analogous to cellular optic nerve models, their shared neuronal origin may indicate IGF-1 as a common protective mechanism against amiodarone-induced oxidative and apoptotic injury. A complete description of the intercellular mechanisms between the RPE and RGC for mitigating amiodarone toxicity remains undetermined. Therefore, additional experimentation is needed to explore apoptosis, oxidative injury, and the protective effects of IGF-1 as a pathophysiologic mechanism of AAON. A continued effort to explore the mechanistic underpinnings of amiodarone toxicity and AAON will aid the clinical challenge of distinguishing AAON from NAION. Correctly identifying early signs of AAON will lessen the risk of serious optic nerve side effects from amiodarone therapy and allow for optimized dosing regiments in patients with severe cardiovascular disease. As knowledge of AAON mechanisms expands, targeted therapeutic agents may ultimately treat or prevent the severe visual side effects of amiodarone toxicity.

## Data Availability

No new data were created or analyzed in this study. Data sharing is not applicable to this article.
